# Supplementary figures and images for: Development of a sensitive real-time quaking-induced conversion (RT-QuIC) assay for application in prion-infected blood
Source: PLoS One. 2023 Nov 2;18(11):e0293845. doi: 10.1371/journal.pone.0293845 (PMC10621866; doi:10.1371/journal.pone.0293845)

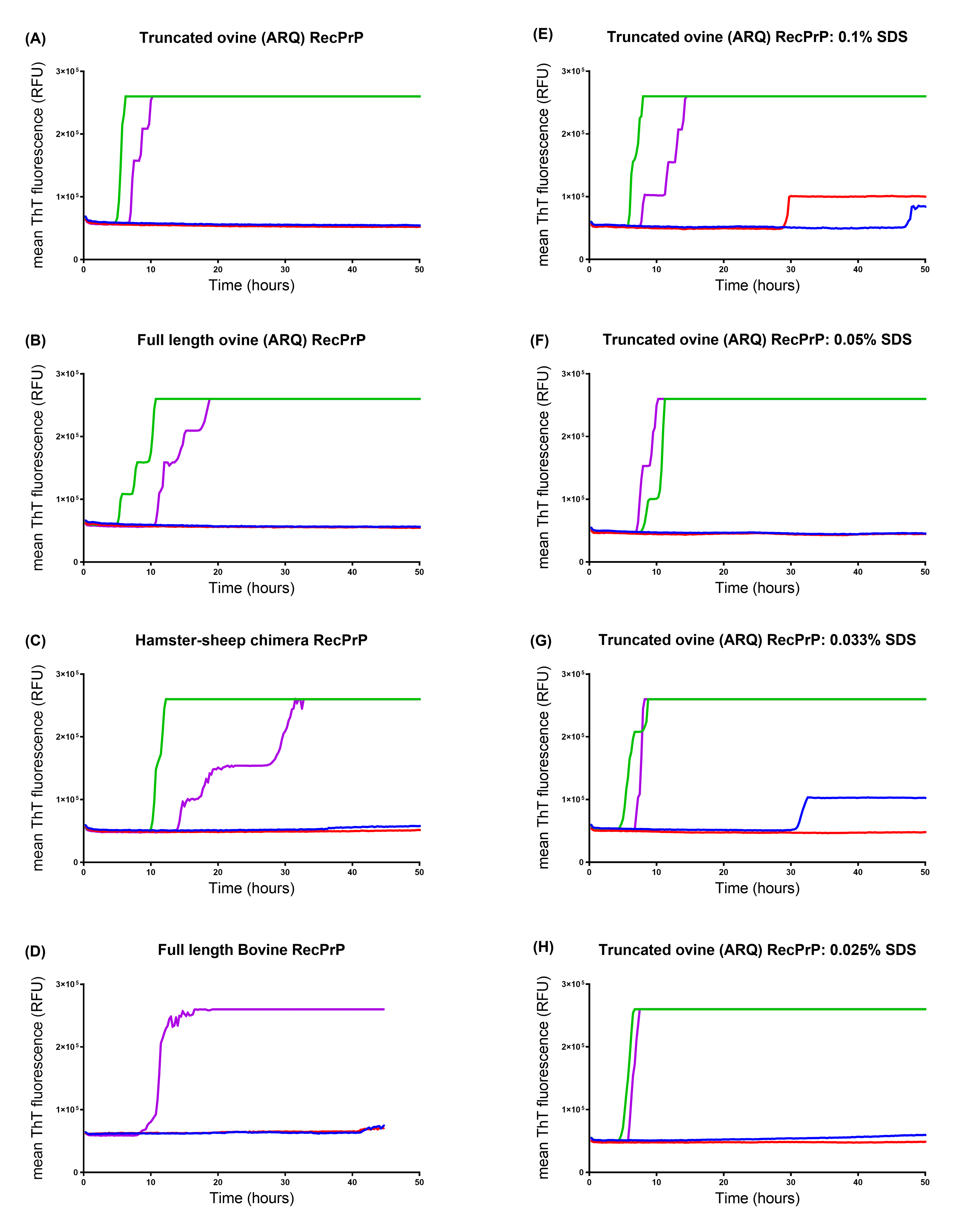

Supplement: S1 Fig — Different substrates were tested by RT-QuIC, including (A) truncated ovine recPrP (amino acids 94–233. PNRP genotype ARQ), (B) full length ovine recPrP (resi 25–233. PNRP genotype ARQ), (C) hamster-sheep recPrP (resi 23–137 Syrian golden hamster PrP followed by resi 141–234 ovine PrP, PNRP genotype ARQ), and (D) full length bovine recPrP (resi 25–241). (E-H) Further experiments were performed to determine the optimal concentration of SDS required for RT-QuIC using truncated ovine recPrP. Based on previous literature, SDS concentrations within the 0.025–0.1% (w/v) range were tested. RT-QuIC reactions were seeded with a 10−4 dilution of BSE-infected sheep brain homogenate (purple) or brain homogenate from mock-infected negative control sheep (red). Unseeded reactions (“mock-seeded” with PBS buffer) are plotted in blue. In most experiments, a 10−4 dilution of 263K scrapie-infected hamster brain tissue was used as a positive control (green). Data points represent the mean ThT fluorescence from n = 4 replicates. (TIF) [file pone.0293845.s001.tif]

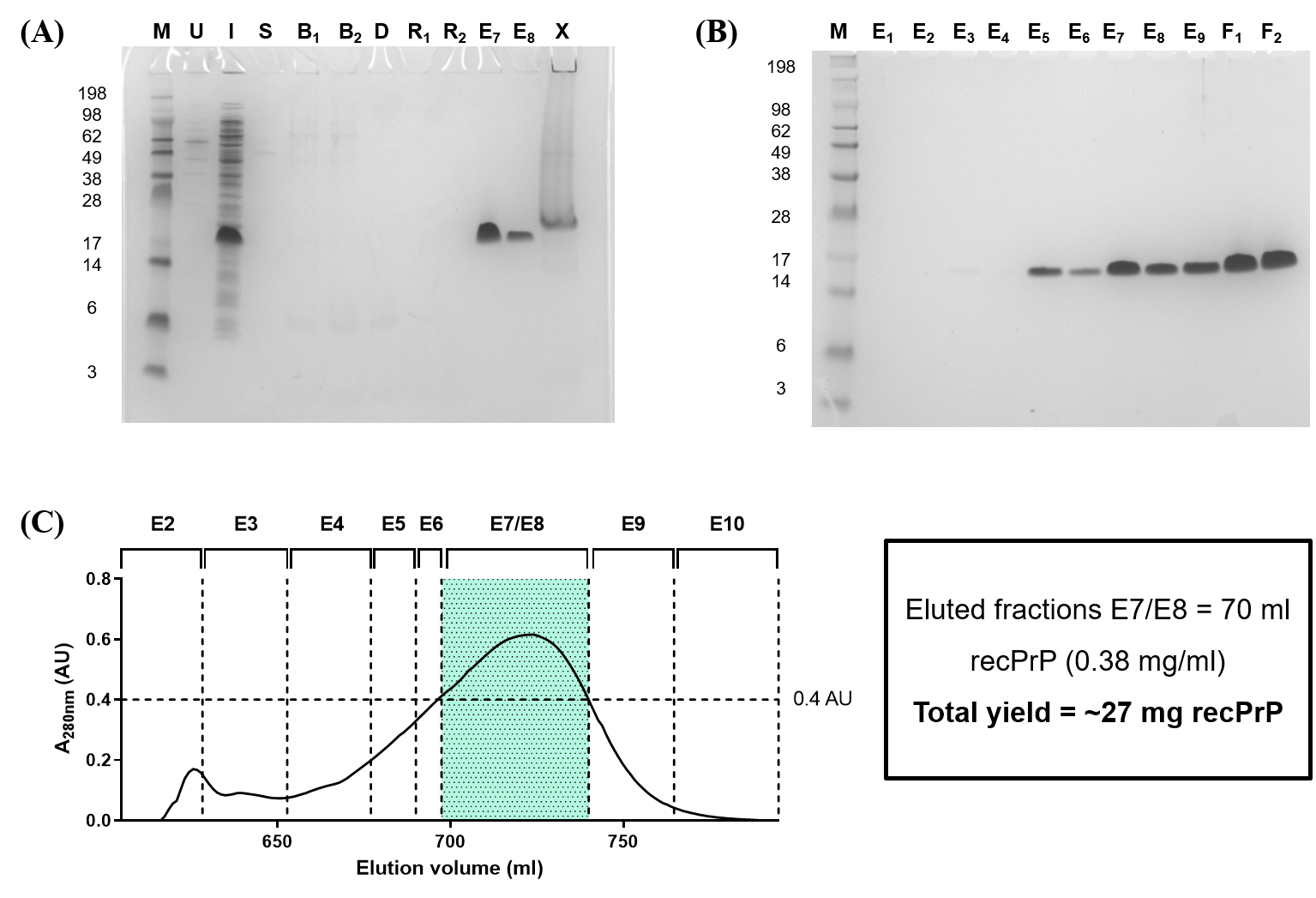

Supplement: S2 Fig — (A) Truncated ovine recPrP was expressed in, and purified from, E. coli Rosetta (DE3) competent cells (Merck). The process was monitored by analysis of fractions on 12% SDS-PAGE gels, with proteins visualised by Coomassie blue: M, molecular mass marker (units in kDa); U, sample from uninduced cells prior to induction with IPTG; I, sample from induced cells; S, sample from supernatant after clarification by centrifugation; B1, sample taken from buffer while bedding resin in column; B2, sample from flow-through while bedding resin; D, sample from denaturing step with guanidine; R1, sample from gradient refolding; R2, sample form isocratic refolding; E7 and E8, eluted fractions containing recPrP, as evidenced by an approximate 17 kDa protein band corresponding to monomeric truncated ovine recPrP; X, sample from resin cleaning step in which any remaining protein was denatured and stripped from the resin. (B) Samples from eluted fractions were assessed for purity by silver staining: E1-E9, samples from gradient elution with imidazole; F1, final dialysed recPrP stock; F2, final recPrP stock passed through 100 kDa MWCO spin filter. (C) Elution of recPrP was monitored by UV. The fraction corresponding to the middle 50% of the elution peak (A280 nm > 0.4 AU) (Fraction E7/E8, green) was pooled to yield 27 mg recPrP. Original, uncropped and minimally-adjusted gel images are provided in another supplementary information file (S1_raw_images). (TIF) [file pone.0293845.s002.tif]

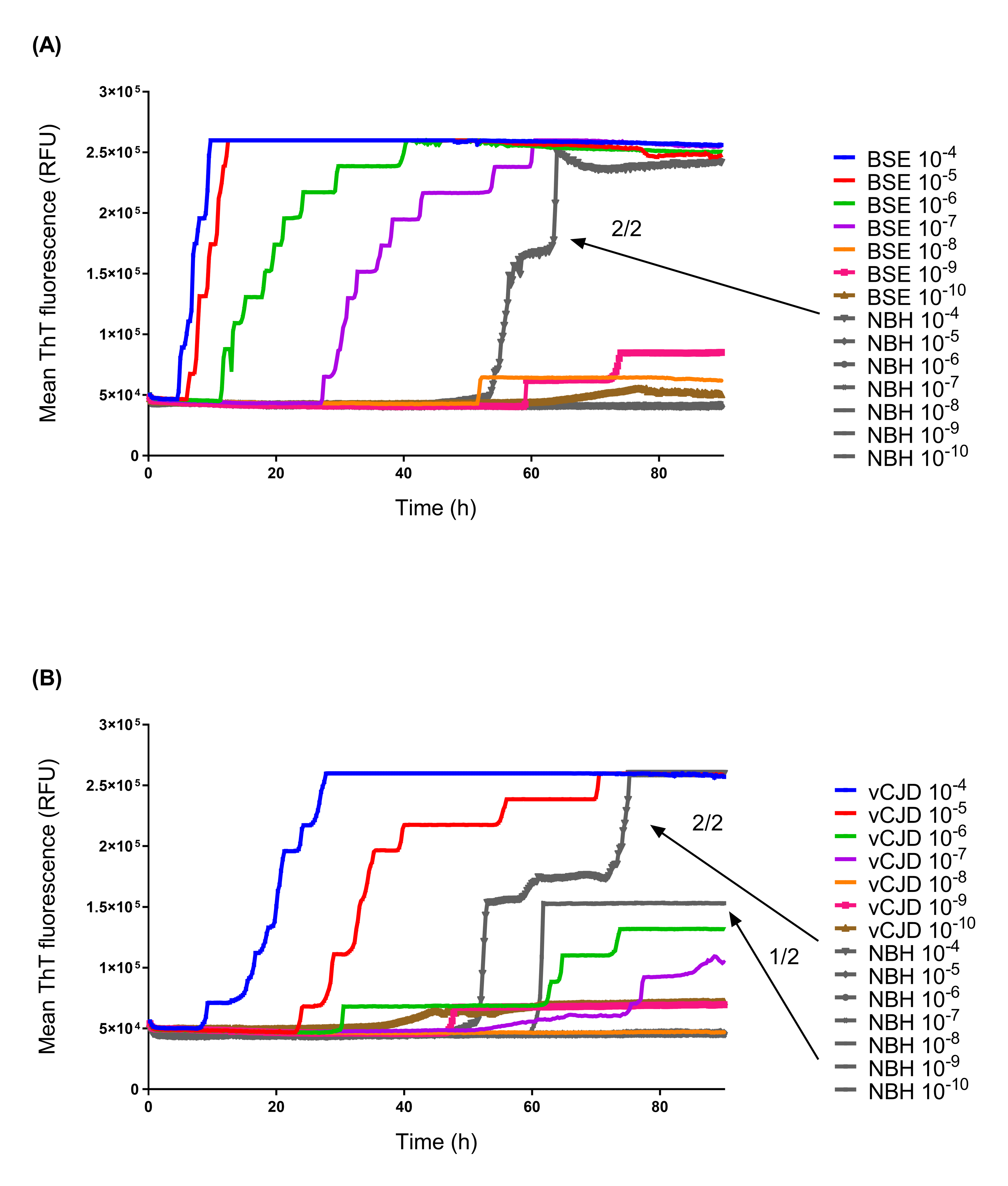

Supplement: S3 Fig — Ten-fold dilutions of (A) a reference BSE-infected sheep brain tissue homogenate and brain tissue from mock-infected negative control sheep (NBH), or (B) a reference vCJD-infected human brain tissue homogenate and negative control human brain tissue. RT-QuIC was performed using truncated ovine recPrP (residues 94–233) as a substrate. Fluorescence measurements were plotted over 90 h. Data points represent the mean ThT fluorescence from n = 10 replicates (for prion-infected brain dilutions), or n = 2 replicates (for negative control brain dilutions). False positives start to appear in 1/2 or 2/2 replicates at given negative control brain dilutions after 50 h (as indicated by arrows). (TIF) [file pone.0293845.s003.tif]

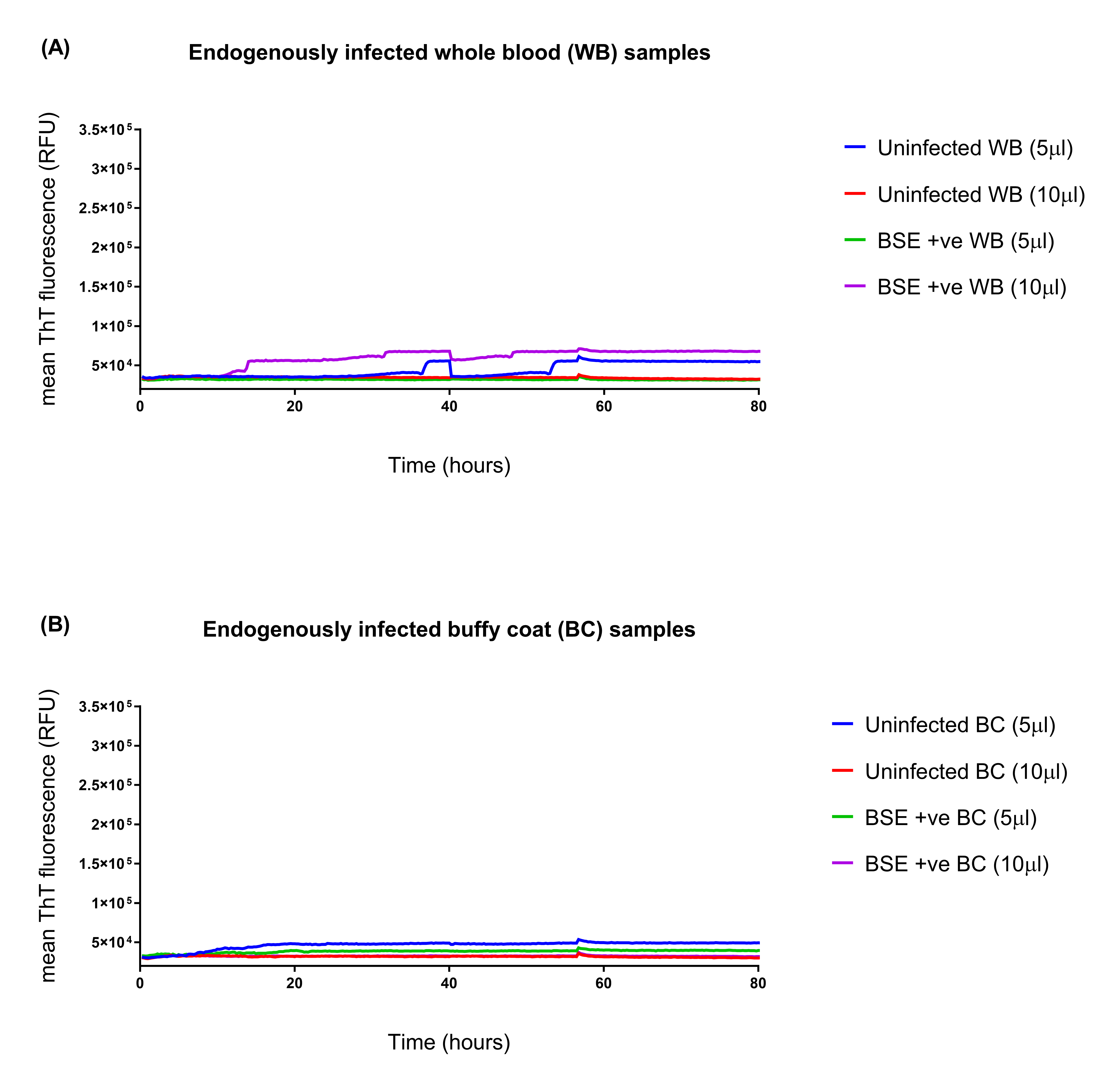

Supplement: S4 Fig — (A) Whole blood (WB) and (B) buffy coat (BC) samples (5–10 μl) from BSE-infected (+ve) sheep (animal ID: N257 at clinical time point) and mock-infected (uninfected) sheep (animal ID: N214 at 0 mpi) were tested by a modified IOME RT-QuIC assay but failed to produce positive results. The mean fluorescence from n = 4 replicate reactions is plotted over a period of 80 h. (TIF) [file pone.0293845.s004.tif]

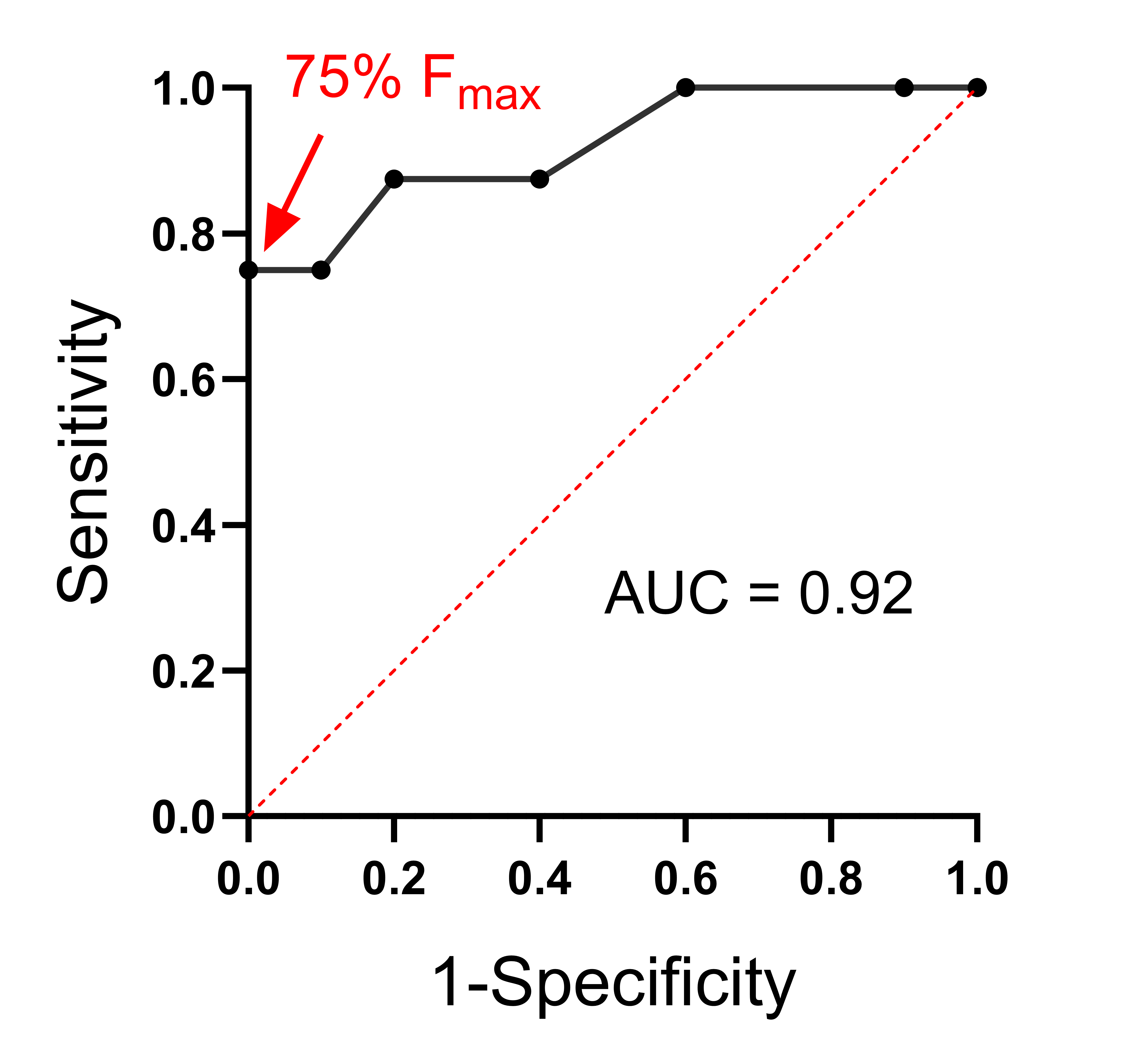

Supplement: S5 Fig — A receiver operating characteristic (ROC) curve and corresponding area under the curve (AUC) was plotted for a representative WB IOME RT-QuIC optimisation experiment testing eight blood samples from known BSE-positive sheep and ten blood samples from known negative sheep (n = 4 replicate reactions/sample). The theoretical sensitivity and specificity of the assay at cut-off (20 h) was plotted for a range of putative threshold values (range 0–100% Fmax). The optimal threshold value was determined to be 75% Fmax, yielding 100% specificity and 75% sensitivity (indicated by red arrow). (TIF) [file pone.0293845.s005.tif]
