# Supplementary material for: Development of a sensitive real-time quaking-induced conversion (RT-QuIC) assay for application in prion-infected blood
Source: PLoS One. 2023 Nov 2;18(11):e0293845. doi: 10.1371/journal.pone.0293845 (PMC10621866; doi:10.1371/journal.pone.0293845)
Supplement: S1 Table — BH, brain homogenate; Proportion of positive replicates defined as: The number of replicate reactions that exceed threshold by cut-off time (50 h)/total number of replicates (n = 10) at a given BH dilution. a Log10 SD50 units (± standard error) present in 2 μg brain calculated according to Spearman-Kärber method, Eqs (1) & (2). b The number of SD50 units per g of brain. (DOCX) [file pone.0293845.s006.docx]

**S1 Table. Summary of RT-QuIC endpoint dilution experiments.**

| **BH Dilution** | **Proportion of positive replicates for reactions seeded with sheep BSE BH** | | | **Proportion of positive replicates for reactions seeded with vCJD BH** | | |
| --- | --- | --- | --- | --- | --- | --- |
|  | **Run 1** | **Run 2** | **Run 3** | **Run 1** | **Run 2** | **Run 3** |
| 10^-4^ | 1 | 1 | 1 | 1 | 1 | 1 |
| 10^-5^ | 1 | 1 | 1 | 0.7 | 0.8 | 0.6 |
| 10^-6^ | 1 | 1 | 1 | 0.2 | 0.1 | 0 |
| 10^-7^ | 0.7 | 0.8 | 0.7 | 0 | 0 | 0 |
| 10^-8^ | 0.1 | 0 | 0.1 | 0.1 | 0 | 0 |
| 10^-9^ | 0 | 0 | 0.1 | 0 | 0.1 | 0 |
| 10^-10^ | 0.1 | 0 | 0 | 0.1 | 0.1 | 0 |
| Log_10_ SD_50_ ^a^ | 7.4 (± 0.21) | 7.3 (± 0.13) | 7.4 (± 0.21) | 5.6 (± 0.25) | 5.6 (± 0.22) | 5.1 (± 0.16) |
| SD_50_/ g ^b^ | 1.26 × 10^10^ | 9.98 × 10^9^ | 1.26 × 10^10^ | 1.99 × 10^8^ | 1.99 × 10^8^ | 6.29 × 10^7^ |

BH, brain homogenate; Proportion of positive replicates defined as: the number of replicate reactions that exceed threshold by cut-off time (50 h)/total number of replicates (n = 10) at a given BH dilution.

^a^ Log_10_ SD_50_ units (± standard error) present in 2 µg brain calculated according to Spearman-Kärber method, equations (1) & (2).

^b^ The number of SD_50_ units per g of brain.
