# Supplementary material for: Development of a sensitive real-time quaking-induced conversion (RT-QuIC) assay for application in prion-infected blood
Source: PLoS One. 2023 Nov 2;18(11):e0293845. doi: 10.1371/journal.pone.0293845 (PMC10621866; doi:10.1371/journal.pone.0293845)
Supplement: S3 Table — BH, brain homogenate; dpi, days post inoculation; NA, not applicable; Proportion of positive mice = the number of mice that scored positive for prion infection (by western blot and/or IHC)/total number of mice in that cohort. a Log10 ID50 units (± standard error) calculated according to Spearman-Kärber method, Eqs (1) & (2). b an estimate of the number of ID50 units per g of brain. (DOCX) [file pone.0293845.s008.docx]

**S3 Table. Summary of tgOvARQ endpoint dilution bioassay.**

| **Dilution of Sh BSE BH** | **Proportion of positive mice** | **Number of positive mice** | **Mean incubation period, dpi (± StDev)** |
| --- | --- | --- | --- |
| 10^-1^ | 0.9 | 7/8 | 329 (± 30) |
| 10^-2^ | 1 | 6/6 | 361 (± 20) |
| 10^-3^ | 1 | 7/7 | 382 (± 29) |
| 10^-4^ | 0.9 | 6/7 | 488 (± 17) |
| 10^-5^ | 0.3 | 2/7 | 560 (± 14) |
| 10^-6^ | 0 | 0/8 | NA |
| 10^-7^ | 0 | 0/8 | NA |
| 10^-8^ | 0 | 0/8 | NA |
| Log_10_ ID_50_ ^a^ | 4.7 (± 0.21) |  | |
| ID_50_/ g ^b^ | 2.51 × 10^6^ |  |  |

BH, brain homogenate; dpi, days post inoculation; NA, not applicable; Proportion of positive mice = the number of mice that scored positive for prion infection (by western blot and/or IHC)/total number of mice in that cohort.

^a^ Log_10_ ID_50_ units (± standard error) calculated according to Spearman-Kärber method, equations (1) & (2).

^b^ an estimate of the number of ID_50_ units per g of brain.
