# Supplementary material for: Development of a sensitive real-time quaking-induced conversion (RT-QuIC) assay for application in prion-infected blood
Source: PLoS One. 2023 Nov 2;18(11):e0293845. doi: 10.1371/journal.pone.0293845 (PMC10621866; doi:10.1371/journal.pone.0293845)
Supplement: S1 Raw images — (PDF) [file pone.0293845.s009.pdf]

Purification gel for E7_E8 truncated sheep recPrP
(SDS-PAGE + coomassie blue stain)

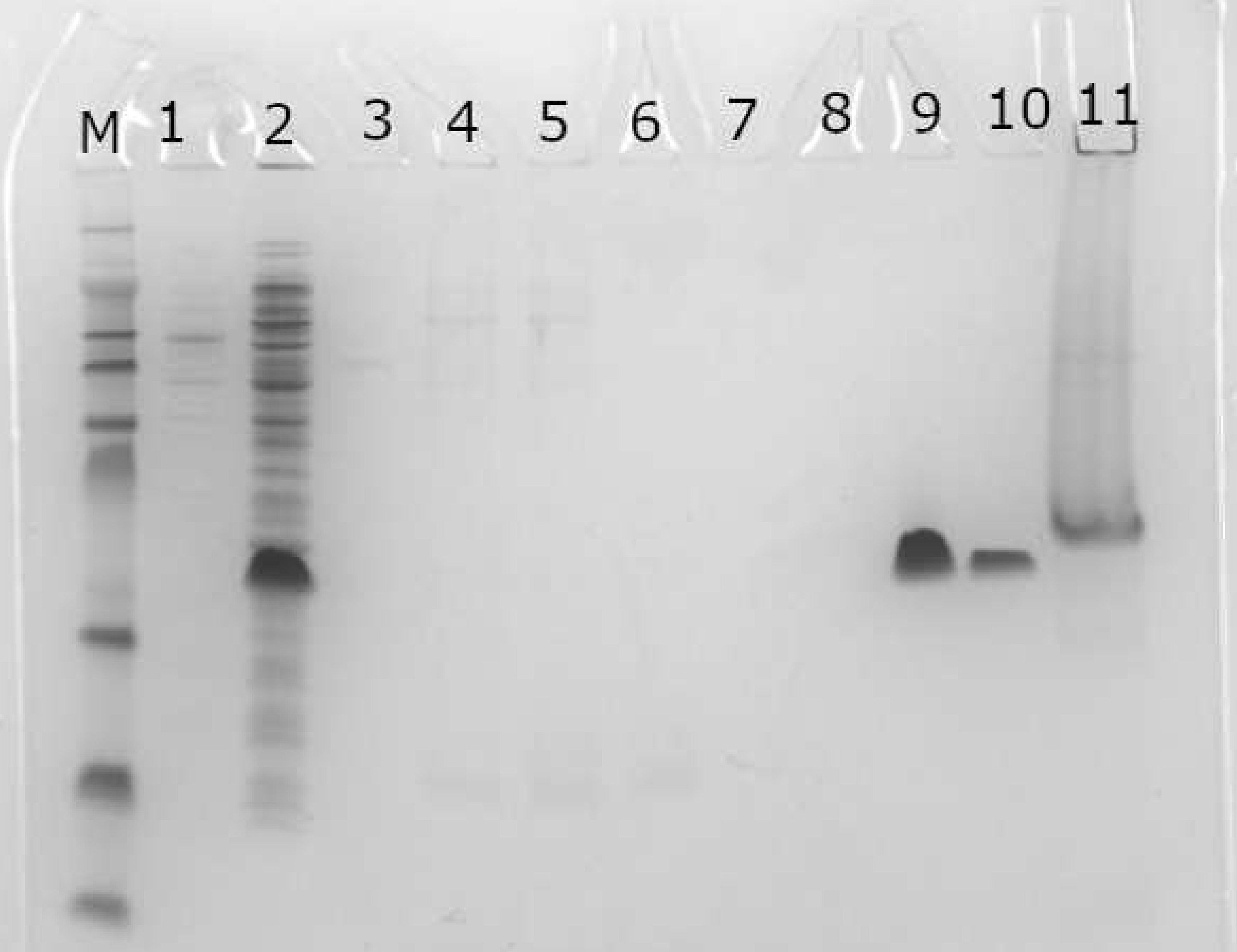


Key: M, molecular weight marker SeeBlue Plus2 pre-stained protein standard (Fisher Scientific); lanes 1-11 represent samples taken during stages of E7_E8 recPrP purificaton: 1, uninduced cells; 2, induced cells; 3, supernatant; 4, resin bedding; 5, resin flow through; 6, denaturing step; 7, gradient refolding; 8, isocratic refolding; 9, elution fraction E7; 10, elution fraction E8; 11, resin wash.
Image captured using Biorad gel doc XR system, Quantity One software (v4.6.7).
Annotations in GIMP (v2.10.34). This raw image was used to generate S2 Fig, panel A.

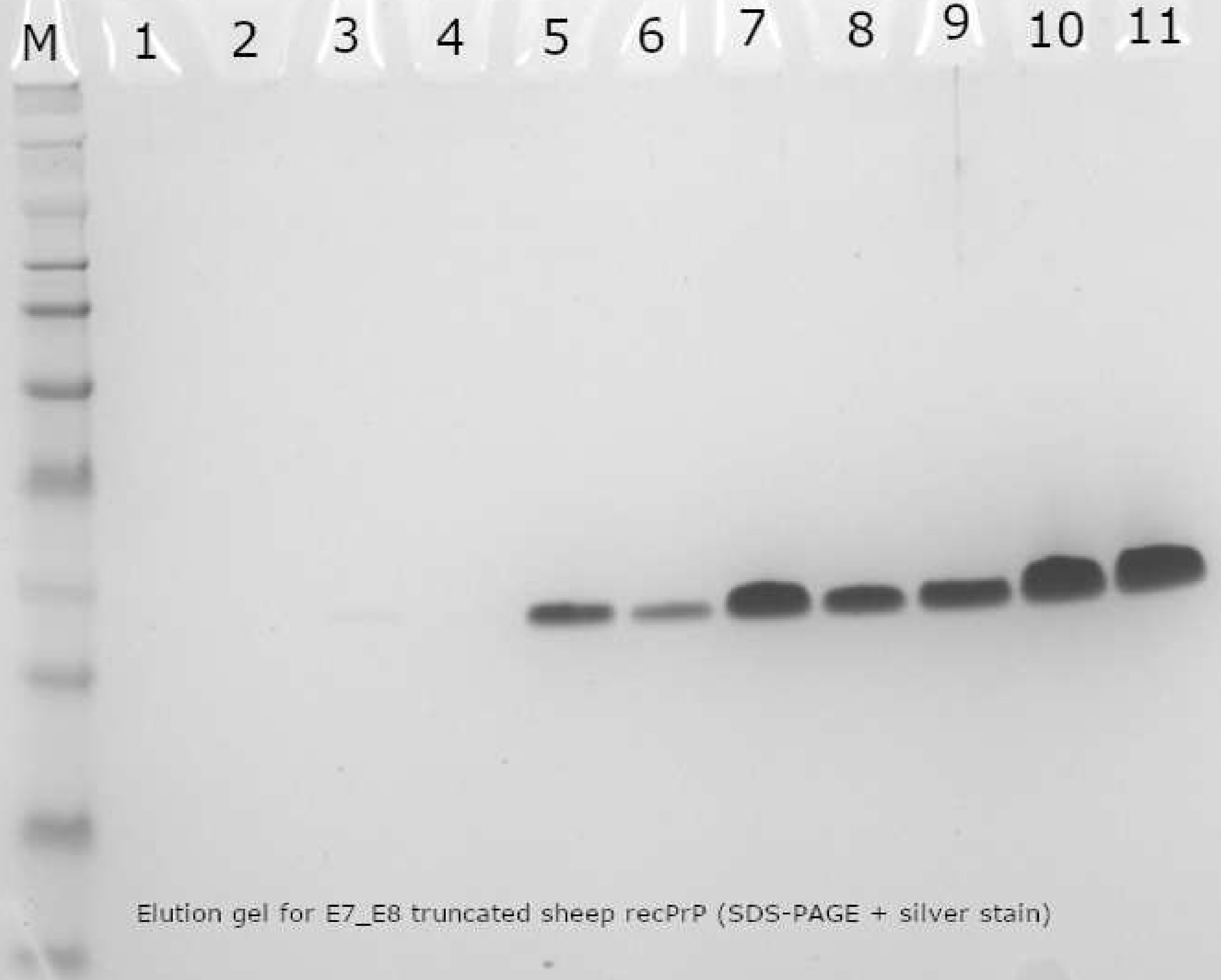


Elution gel for E7_E8 truncated sheep recPrP (SDS-PAGE + silver stain)

Key: M, molecular weight marker, SeeBlue Plus2 pre-stained protein standard (Fisher Scientific). Lanes 1-9 represent eluted fractions E1-E9; 10, dialysed recPrP stock; 11, filtered recPrP stock. Image captured using Biorad gel doc XR system, Quantity One software (v4.6.7). Annotations in GIMP (v2.10.34). This raw image was used to generate S2 Fig, panel B.
